# Association between acculturation, obesity and cardiovascular risk factors among male South Asian migrants in the United Arab Emirates – a cross-sectional study

**DOI:** 10.1186/s12889-015-1568-x

**Published:** 2015-02-28

**Authors:** Syed M Shah, Tom Loney, Salma Al Dhaheri, Hassan Vatanparast, Iffat Elbarazi, Mukesh Agarwal, Iain Blair, Raghib Ali

**Affiliations:** Institute of Public Health, College of Medicine and Health Sciences, United Arab Emirates University, Al Ain, United Arab Emirates; Ambulatory Health Services, SEHA, Al Ain, United Arab Emirates; School of Public Health, College of Nutrition and Pharmacy, University of Saskatchewan, Saskatchewan, Canada; Department of Pathology, College of Medicine and Health Sciences, United Arab Emirates University, Al Ain, United Arab Emirates

**Keywords:** Acculturation, Adiposity, Cardiovascular diseases, Obesity, Transients and migrants, United Arab Emirates

## Abstract

**Background:**

Approximately 65% of the United Arab Emirates (UAE) population are economic migrants from the low- and middle-income countries of South Asia. Emerging evidence suggests that expatriate populations from low or middle-income countries that migrate to high-income countries acculturate their lifestyle with the obesogenic behaviours of the host country. Previous research has focussed on migrant populations in the United States. The objective of this study was to assess the prevalence of obesity and explore the relationship between years of residency (surrogate measure for acculturation) and obesity among South Asian (from India, Pakistan and Bangladesh) male immigrants residing in the UAE.

**Methods:**

A random sample of 1375 males was recruited from a mandatory residency visa health screening centre in Abu Dhabi (UAE). Employing a cross-sectional design, participants completed an interviewer-led adapted version of the World Health Organisation STEPS questionnaire, and anthropometric and blood pressure measurements were collected. Glycated haemoglobin (HbA1c) was measured in a random sub-sample (n = 100). Logistic regression was used to determine risk factors for being classified as obese, and to assess the relationship between years of residency and adiposity.

**Results:**

The overall prevalence of body mass index-derived overweight and obesity estimates and waist-to-hip-derived central obesity rates was 615 (44.7%) and 917 (66.7%) males, respectively. Hypertension was present in 419 (30.5%) of the sample and diabetes in 9 (9.0%) of the sub-sample. Living in the UAE for six to 10 years or more than 10 years was independently associated with being classified with central obesity (adjusted odds ratio [AOR] 1.63 95% confidence intervals [CI] 1.13 - 2.35, *p* < 0.008; AOR 1.95 95% CI 1.26 - 3.01, *p* < 0.002; respectively) compared to residing in the UAE for one to five years.

**Conclusions:**

Our study revealed a high prevalence of overweight, central obesity and hypertension amongst a young South Asian male migrant population in the UAE. Study findings suggest a diminished ‘Healthy Migrant Effect’ with increased years of residency possibly due to greater acculturation and a transition in lifestyle behaviours. Health initiatives targeting the maintenance of a healthy body size, coupled with regular assessments of glucose control and blood pressure are urgently required in this population.

## Background

According to the World Health Organization (WHO), non-communicable chronic diseases (NCDs) such as cardiovascular disease, cancer, chronic respiratory disease and diabetes cause 60% of all deaths globally [1]. By 2030, the global cost of NCDs will reach $47 trillion [2]. Moreover, 80% of this mortality occurs in low- and middle-income countries [1]. Numerous studies have shown that South Asian populations (e.g. India, Pakistan, Bangladesh, Sri Lanka, and Nepal) contribute the highest proportion of the global burden of cardiovascular diseases [3,4]. In addition, risk factors for NCDs such as central obesity, hypertension and insulin resistance develop at a lower age in South Asians than other ethnic groups [5,6]. Moreover, South Asian immigrants overseas have a three to five-fold increase in the risk for myocardial infarction and cardiovascular death compared with other ethnic groups [7,8].

Obesity is a major contributor to cardiovascular disease and hence, it is a leading risk factor for adult mortality in both developed and developing countries [9]. People from Asia have a higher risk of type 2 diabetes and cardiovascular disease at lower levels of body mass index (BMI) [10]. Thus, in South Asians migrants, substantially lower obesity levels show an equivalent amount of dyslipidemia and dysglycemia when compared to BMI-matched White Europeans [11]. Furthermore, immigrant populations relocating from low- and medium-income countries to a higher income country are at a greater risk of weight gain, particularly males [12]. The ‘Healthy Migrant Effect’ is a phenomenon observed in epidemiology where newly arrived migrants tend to be healthier than the host population due to a positive selection bias that a formal migration process and residency health checks require. Emerging evidence suggests that the ‘Healthy Migrant Effect’ may diminish with increased years of residency due to lifestyle and nutrition transition associated with acculturation i.e. the complex, gradual exchange of immigrants’ original attitudes and behaviours to the obesogenic behaviours in the host culture. A recent systematic review synthesised data from nine cross-sectional studies of Mexican, El Salvador, Puerto Rico, Thailand/Laos Hmong, Korean and Soviet Union immigrants residing in the United States. Findings showed a positive association between higher acculturation and body mass index in males [12].

In the past 40 years, the United Arab Emirates (UAE) has achieved significant economic and industrial growth. The rising affluence of the UAE population with its accompanying increased caloric intake, and decreased physical activity has rapidly increased the prevalence of obesity [13,14]. Consequently, the UAE ranks sixth in a recent indexing of the top 10 ‘heaviest’ countries in the world (ranking determined by number of adults per tonne of human biomass) [15]. The expatriate population accounts for approximately 89 percent of UAE population and approximately two-thirds of all the immigrants are South Asian [16]. The UAE population has increased substantially over the past four decades and this is primarily due to the high net inward migration of expatriate workers from South Asia (UAE population estimates: 287,000 in 1971, 4.1 million in 2005, 8.3 million in 2010). Abu Dhabi is the largest of the seven UAE emirates with an estimated population of 2.3 million, of which over half of the population are expatriate males aged 20-59 years. All expatriate workers seeking employment in the UAE are required by federal law to undergo a health and communicable disease screening test at a government visa screening centre before receiving a residency permit. In 2012, 181,231 male immigrants visited the visa screening centre (Disease Prevention and Screening Centre, Preventive Medicine Department, Health Authority Abu Dhabi) in the city of Al Ain (second largest city in Abu Dhabi Emirate) to obtain or renew a visa [17]. Majority of these workers are low-paid, semi- or unskilled, without long-term job security, living alone or in shared accommodation, and separated from their families, they often suffer from stress, anxiety and depression [18]. A dearth of information exists on the prevalence of overweight and obesity among South Asian immigrants in the UAE. The aim of this study was to assess the prevalence of obesity and explore the relationship between years of residency (surrogate measure for acculturation) and obesity among South Asian (from India, Pakistan and Bangladesh) male immigrants residing in the UAE.

## Methods

### Study design and ethics

The study used a cross-sectional descriptive design. Ethical approval was obtained from both the Al Ain Medical District Human Research Ethics Committee, and the Abu Dhabi Health Services Company’s Research Committee.

### Study sample and setting

The study took place between January and June 2012. All expatriate workers seeking employment in the UAE are screened for communicable diseases, such as tuberculosis (by chest X-ray) and human immunodeficiency virus (by serology), before acquiring residence status. We invited every third migrant worker (from India, Pakistan and Bangladesh) who visited the Preventive Medicine Department in Al Ain for a health examination to obtain a new or renew an existing visa to participate in the study. Of the 1800 eligible participants, 1375 (76.4% response rate) participated in the study.

### Measures

#### Health and lifestyle questionnaire

The informed consent forms and questionnaires used in the study were written in the native languages of the workers, i.e., Urdu and Bangali. The questionnaire was initially developed in English, and then forward translated into Urdu and Bengali. It was then pretested in the pilot study and finalised after necessary amendments. The questionnaire comprised an adapted version of WHO STEPwise approach to Surveillance, “STEPS”, developed by WHO for the measurement of NCD risk factors at the country level [19]. Due to the low literacy rates among the South Asian expatriate population in the UAE, all questionnaires were completed during an interview with a native Urdu or Bengali speaking researcher. The questionnaire interview collected information that included demographic characteristics, personal and family and disease history, history of current and past consumption of cigarettes, alcohol, other forms of tobacco (history of exposure to second hand tobacco), home country residence type (rural, urban, semi-urban), occupation, monthly salary in UAE dirhams (AED), years of residency in the UAE, accommodation type, and physical activity. Information related to the diagnosis and treatment history of hypertension and diabetes was also collected.

We classified monthly earning into bottom quartile (AED <900 per month), second quartile (AED 900 to <1200), third quartile (AED 1200 to 2000), and top quartile (>2000). Using STEPS [19] questions on tobacco use, we classified subjects as current smokers if they answered yes to the question: *"Do you smoke cigarettes daily?"*. Former smokers were defined if they had ever smoked at least 100 cigarettes during their lifetime. Ever smokers included current and former smokers. The variable relating to alcohol consumption was based on the STEPS question: *"Did you drink any alcohol during the last 12 months?"* The variables used for the analysis were current smoking and alcohol consumption during the last year. Information on physical activity was obtained using the International Physical Activity Questionnaire (IPAQ-short version) which measures the frequency (days per week), and duration (minutes per day) of moderate- and vigorous-intensity physical activity, in bouts of at least 10 minutes during the past 7-day period, globally in all contexts of daily life [20]. The IPAQ also assesses the time spent walking (≥10 minutes duration) during the past 7 days and duration of walking within a given day in the last seven days was recorded to identify people who walked for at least 30 minutes each day.

#### Anthropometric and blood pressure measurements

Researchers were trained using a standard protocol to obtain anthropometric measurements. Body mass was measured (to the nearest 0.1 kg) using a calibrated electronic scale equipped with a mounted stadiometer that measured height to the nearest 0.1 cm (SECA Hamburg, Germany). Body mass and height measurements were completed with the participant wearing light clothing without shoes and standing motionless. Waist and hip circumference were measured using a flexible non-stretch nylon tape measure (SECA Hamburg, Germany) with subjects wearing light clothing. Waist circumference was measured midway between the lower rib margin and the top of the iliac crest at the end of a gentle expiration to the nearest 0.1 cm. Hip circumference was measured at the point of maximal protrusion of the gluteal muscles also to the nearest 0.1 cm. Body mass index (BMI) was calculated as body mass in kilograms divided by height in metres squared. The WHO cut-offs were used to classify subjects as overweight (25.0-29.9 kg/m^2^) or obese (30.0 kg/m^2^) [21]. Waist circumference (WC) ≥ 94.0 cm and waist-to-hip ratio (WHR; waist in cm/hip circumference in cm) ≥0.90 was used to define central obesity [22].

We followed 1999 World Health Organization/International Society of Hypertension guidelines to measure blood pressure [23]. Following 10 minutes of rest in a seated position, resting brachial blood pressure was measured with a calibrated automated sphygmomanometer (Omron HEM-705cp Intellisense Blood Pressure Monitor). Triplicate measurements were taken, three minutes apart on the right arm, and the mean of the last two readings was used for analyses. We classified participants as hypertensive if they had a measured systolic blood pressure above 140, diastolic blood pressure above 90, or if they reported taking medications that treat hypertension [24]. In addition, we collected a non-fasting venous blood sample to measure glycated haemoglobin (HbA1c) levels in a random sub-sample (n = 100) of the study population. The WHO cut-off of HbA1c ≥ 6.5% was used to indicate the presence of diabetes mellitus in the sub-sample.

### Statistical analysis

Data files were created in Microsoft Access software. After cleaning and processing, data were imported into Stata SE version 11.0 (StataCorp LP, College Station, TX) for analysis. In descriptive analyses, percentages means and 95% confidence intervals (CI) were calculated. Chi-squared test were conducted for categorical variables and analyses of variance was used for continuous variables. We used bivariate and multivariate models and for the logistic regression model we used a forward selection stepwise process to examine the association between socio-demographic, lifestyle and acculturation (indexed by years of residency) with the odds of overweight and obesity (based on BMI categories) and central obesity (waist-to-hip ratio). Alpha was set at 0.05. In our multivariable model we used age and income as continuous variable.

We also evaluated the effects of acculturation on other cardiovascular risk factors i.e. hypertension, diabetes and smoking. Participants who either self-reported doctor-diagnosed diabetes or who were taking medications that treat diabetes or their A1C level was greater or equal to 6.5 were classified as diabetes.

## Results

Out of 1800 eligible participants 1375 (76.4% response rate) participated in the study. Of the participants 433 were Indian, 383 were Pakistani and 559 were Bangladeshi. The mean age of the study population was 34.0 (95% CI: 33.4, 34.5) years. Table 1 shows the general characteristics of the study population. Compared to Indian and Pakistani counterparts, Bangladeshi males were younger. The overall monthly average income in AED (1 USD = 3.7 AED) was 1828 (95% CI 1722, 1944). A high proportion of Bangladeshi subjects (60.4%) earned less than 1000 AED per month compared to their Indian (20.6%) and Pakistani (32.3%) counterparts. Bangladeshi subjects were also less educated. Only 13.5% had college or university level education compared to Indians (31.7%) and Pakistanis (16.4%). Of the study participants, 68% (95% CI 66.1, 71.1) had migrated from rural villages in their home country. Overall, the majority (70%) of subjects were married with on average three children. Nearly all (85%) of those who were married lived away from their families. Half (52.2%) of the study sample shared a rented accommodation with others while the remainder lived with their sponsor (13.4%), in a single accommodation (11.1%), in labour camps (12.2%) or with family members (11.1%). Over half (55.1%) of participants had been living in UAE for more than six years. The most common occupational categories were driver (23.1%), labourer (17.1%), agricultural worker (17.1%), construction worker (12.5%) and salesman (5.7%).

**Table 1:** **Characteristics of the South Asian Immigrants in Abu Dhabi, UAE 2012**

	**India (n = 433)**	**Pakistan (n = 383)**	**Bangladesh (n = 559)**
**Characteristics**	**Mean (± SD)**	**Mean (± SD)**	**Mean (± SD)**
Age (mean) years	36.3 (±10.1)	34.8 (±10.7)	31.7 (±8.5)
Education (%)			
None	3.1	23.9	12.9
Primary or middle	23.3	31.4	46.0
Secondary or high school	36.9	28.3	27.6
College or university	36.7	16.4	13.5
Income in Emirati Driham (AED) per month, median	1500	1500	1000
Immigrated from a rural village (%)	64.3	56.7	83.2
Married (%)	78.5	75.3	60.1
Do not live with immediate family (%)	88.6	88.4	81.0
Type of accommodation (%)			
Shared with non-relatives	51.4	41.1	58.6
Shared with family	10.5	15.0	7.8
Single accommodation	13.3	11.1	8.9
Live with sponsor	13.3	16.7	12.2
Live in a labor camp	11.5	16.1	12.4
Years in UAE (mean)	10.3 (±8.6)	11.7 (±9.9)	8.2 (±6.8)
Occupation (%)			
Driver	21.5	33.9	19.2
Laborer	14.1	12.8	20.3
Construction worker	12.6	8.3	13.1
Agriculture worker	8.2	25.0	20.5
Salesman	12.6	3.1	3.3
Professional, office worker	10.8	6.7	3.6
Business shop keeper	5.4	4.2	4.0
Hospitality worker (cook, waiter)	8.2	0.8	6.5
Tailor	3.6	1.9	6.8
Other	3.1	3.3	2.7
Family history of hypertension (%)	14.8	15	23.2
Family history of diabetes (%)	15.9	8.1	8.9
Body mass index (kg/m^2^), mean	25.1 (±4.8)	25.4 (±5.2)	23.9 (±4.1)
Waist-to-hip ratio, mean	0.93 (±0.08)	0.93 (±0.09)	0.92 (±0.31)
Waist circumference (cm), mean	89.7 (±10.3)	92.7 (±13.7)	85.9 (±10.5)
Smoking (%)	34.3	35.8	49.1
Smokeless tobacco (%)	5.2	11.1	15.6
Exposure to 2ndhand tobacco (%)	40.7	49.7	46.7
Drinking alcohol (ever %)	20.1	3.1	7.8
Walk 7 days a week for at least 30 minutes a day (%)	81.1	85.9	77.2
Reported vigorous physical activity ≥1 day in during last 7 days (%)	17.1	18.8	18.6
Reported moderate physical activity ≥1 day in during last 7 days (%)	20.5	18.9	37.0
Blood pressure (BP) measurement (never %)	57.3	62.7	68.8
Hypertension (BP ≥ 140/90 mm Hg or using antihypertensive drugs (%)	34.5	28.2	28.8
Physician diagnosed diabetes (%)	10.8	6.5	6.8
Glycosylated hemoglobin or HbA1c ≥ 6.5%	5.8 (±1.1)	5.7 (±1.7)	5.9 (±1.3)

A higher proportion of Bangladeshi subjects (23.2%) reported a family history of hypertension compared to Indians (14.8%) and Pakistanis (15.0%). A family history of diabetes was significantly more prevalent among Indians (15.9%) compared to Pakistanis (8.1%) and Bangladeshi (8.9%) participants. Indians had a higher mean BMI (31.5 kg/m^2^) compared to Pakistanis (26.6 kg/m^2^) and Bangladeshis (26.2 kg/m^2^). Bangladeshis had lower waist circumference (85.6 cm) and waist-to-hip ratio (0.92 cm) but reported a high rate of smoking (49.1%) as compared to their Indian (34.3%) and Pakistani (35.8%) counterparts. Overall, 9.0% of the sub-sample (n = 99) were diabetic (HbA1c ≥6.5%) and most of these reported having a physician diagnosis of diabetes. The prevalence of hypertension was 30.5% despite this being a young population of South Asian males. Nearly two-thirds (61.6%) of the sample reported that they had never had their blood pressure measured. Among the study population, 44.4% of participants with diabetes and 76.0% of those with hypertension were not aware of their status. Overall a small proportion of the study participants reported moderate 26.7% (95% CI 24.4 - 29.1) and vigorous, 18.2% (95% CI 16.1 - 20.2) physical activity. Self-reported vigorous and moderate physical activity was particularly low among drivers (13.6%, 14.2%), shopkeepers and business men (13.3%, 15.0%), and tailors (4.3%, 7.1%).

The overall crude prevalence of overweight and obesity in South Asian immigrants were 35.4% (95% CI 32.8%, 37.9%), and 9.4% (95% CI 9.2%, 9.5%), respectively. The crude prevalence of central obesity was 63.4% (95% CI 60.8%, 65.9%). The crude prevalence of overweight and obesity was highest among Pakistanis, 52.5% (95% CI 47.3%, 57.5%), followed by Indians, 47.4% (95% CI 42.7%, 52.2%) and Bangladeshis, 37.4% (95% CI 33.4%, 41.6%). The overall crude prevalence of central obesity varied by nationality, 66.7% (95% CI 62.8%, 71.1%) in Indians, 64.7% (95% CI 59.7%, 69.5%) in Pakistanis, and 59.9% (95% CI 55.7%, 64.0%) in Bangladeshis.

**Figure 1:**
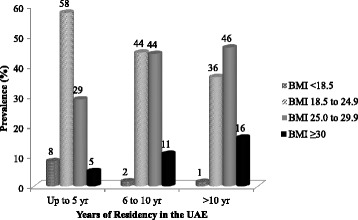
**Prevalence of body mass index-derived estimates of underweight, normal weight, overweight and obesity by years of residency in the UAE for South Asian immigrants.**

Figure 1 illustrates the prevalence of underweight, normal weight, overweight and obesity by the duration of residency in South Asian immigrants. The prevalence of underweight decreased from 8% among those who had been in UAE for up to five years, to 1% among those who had been in UAE for more than 10 years. Prevalence of overweight and obesity was significantly (*p* < 0.05) higher (62%) among those who had been in UAE for more than 10 years.

Table 2 presents the prevalence of overweight, obesity and central obesity and the factors significantly (*p* < 0.05) associated with being overweight or obese (BMI ≥25.0). In un-adjusted analyses these factors included nationality, greater age, greater monthly salary, education, working as a driver, salesman, office worker, having a business or working as shop-keeper, or tailor, type of current accommodation, urban residence in their country of origin, being married, longer duration of residency in UAE and being hypertensive.

**Table 2:** **Prevalence and un-adjusted odds ratios of factors associated with overweight/obesity and central obesity, in male South Asian Immigrants, (n = 1,375) Al Ain, Abu Dhabi, UAE, 2012**

		**Overweight**				**With central obesity**		
		**BMI ≥25.0**	**Crude OR**	***p*** **value**		**Waist-to-hip ratio ≥0.90 cm**	**Crude OR**	***p*** **value**
Nationality	All	n (%)	(95% CI)		All	n (%)	(95% CI)	
India	432	205 (47.4)	1.51 (1.16 - 1.94)	0.002	433	289 (66.7)	1.34 (1.03 - 1.74)	0.028
Pakistan	383	201 (52.5)	1.84 (1.42 - 2.40)	0.000	383	248 (64.7)	1.23 (0.93, - 1.61)	0.135
Bangladesh	558	209 (37.5)	1.0		559	335 (59.9)	1.0	
Age group (n,%)								
18-35 years	809	282 (34.8)	1.0		810	417 (51.5)	1.0	
36-45 years	307	182 (59.3)	2.72 (2.07 - 3.56)	0.000	307	244 (79.5)	3.65 (2.67 - 4.97)	0.000
46+ years	199	124 (62.3)	3.08 (2.24 - 4.26)	0.000	200	174 (87.0)	6.31 (4.08 - 9.74)	0.000
Monthly salary in UAE Dirhams (AED), n, %								
Botom quartile	370	103 (27.8)	1.0		371	198 (53.4)	1.0	
Second quartile	290	108 (37.2)	1.54 (1.11 - 2.14)	0.010	290	176 (60.7)	1.35 (0.99 - 1.84)	0.060
Third quartile	399	219 (54.9)	3.15 (2.33 - 4.26)	0.000	399	275 (68.9)	1.94 (1.44 - 2.60)	0.000
Top quartile	314	185 (58.9)	3.72 (2.70 - 5.12)	0.000	315	223 (70.8)	2.11 (1.54 - 2.91)	0.000
Education (n,%)								
No formal schooling	172	55 (31.9)	1.0		173	98 (56.6)	1.0	
Primary or middle	473	196 (41.4)	1.50 (1.04 - 2.18)	0.030	473	301 (63.6)	1.34 (0.94 - 1.91)	0.106
Secondary or high school	585	283 (48.4)	1.99 (1.39 - 2.85)	0.000	586	387 (66.0)	1.49 (1.05 - 2.10)	0.024
College or university	139	80 (57.5)	2.88 (1.81 - 4.59)	0.000	139	83 (59.7)	1.13 (0.72 - 1.78)	0.586
Occupation (n, %)								
Driver	317	190 (59.9)	3.29 (2.31 - 4.71)	0.000	317	220 (69.4)	1.94 (1.37 - 2.76)	0.000
Laborer	234	73 (31.2)	1.0		234	137 (58.5)	1.21 (0.84 - 1.74)	0.301
Construction worker	171	61 (35.7)	1.22 (0.80 - 1.85)	0.345	172	103 (59.9)	1.28 (0.86 - 1.91)	0.222
Agriculture worker	235	79 (33.6)	1.11 (0.75 - 1.64)	0.576	236	127 (53.8)	1.0	
Salesman	79	38 (48.1)	2.04 (1.21 - 3.44)	0.007	79	52 (65.8)	1.65 (0.97 - 2.81)	0.063
Professional, office worker	95	52 (54.7)	2.66 (1.63 - 4.34)	0.000	95	63 (66.3)	1.68 (1.02 - 2.77)	0.038
Business/shop keeper	60	40 (66.7)	4.41 (2.41 - 8.07)	0.000	60	49 (81.7)	3.82 (1.89 - 7.72)	0.000
Hospitality worker	71	28 (39.4)	1.43 (0.82 - 2.49)	0.197	71	47 (66.2)	1.68 (0.96 - 2.92)	0.066
Tailor	70	36 (51.4)	2.33 (1.35 - 4.02)	0.002	70	47 (67.1)	1.75 (1.00 - 3.07)	0.049
Other	40	18 (45.0)	1.80 (0.91 - 3.57)	0.090	40	26 (65.0)	1.59 (0.79 - 3.20)	0.191
Type of accommodation								
Shared with non-relatives	716	314 (43.8)	1.63 (1.14 - 2.33)	0.007	717	459 (64.0)	1.40 (0.99 - 1.97)	0.053
Shared with family	152	82 (53.9)	2.45 (1.55 - 3.86)	0.000	152	91 (59.9)	1.17 (0.75 - 1.83)	0.479
Single accommodation	153	89 (58.2)	2.91 (1.84 - 4.59)	0.000	153	113 (73.8)	2.22 (1.38 - 3.56)	0.001
Live with sponsor	184	76 (41.3)	1.47 (0.95 - 2.28)	0.083	184	114 (61.9)	1.28 (0.83 - 1.96)	0.253
Live in a labor camp	167	54 (32.3)	1.0		168	94 (55.9)	1.0	
Home country setting								
Rural	930	381 (40.9)	1.0		932	575 (61.7)	1.0	
Urban or semi-urban	426	228 (53.5)	1.66 (1.32 - 2.09)	0.000	426	285 (66.9)	1.25 (0.99 - 1.59)	0.065
Length of stay in UAE								
1 to 5 years	546	187 (34.2)	1.0		547	287 (54.5)	1.0	
6 to 10 years	257	139 (54.1)	2.26 (1.67 - 3.06)	0.000	257	182 (70.8)	2.19 (1.60 - 3.02)	0.000
>10 years	413	259 (62.7)	3.23 (2.47 - 4.21)	0.000	414	346 (83.6)	4.61 (3.38 - 6.28)	0.000
Marital status								
Single	411	118 (28.7)	1.0		412	178 (43.2)	1.0	
Married	962	497 (51.7)	2.65 (2.07 - 3.40)	0.000	963	694 (72.1)	3.39 (2.66 - 4.31)	0.000
Smoking ever								
Never	826	361 (43.7)	1.0		827	510 (61.7)	1.0	
Ever	547	254 (46.4)	1.12 (0.89 - 1.38)	0.319	548	362 (66.1)	1.21 (0.96 - 1.51)	0.098
Smokeless tobacco (n, %)								
Never	1160	514 (44.3)	1.0		1162	735 (63.2)	1.0	
Ever	150	76 (50.7)	1.29 (0.91 - 1.81)	0.142	150	97 (64.7)	1.06 (0.74 - 1.52)	0.735
Drinking alcohol								
Never	1118	490 (43.8)	1.0		1119	696 (62.2)	1.0	
Ever	129	66 (51.2)	1.34 (0.93 - 1.93)	0.113	130	86 (66.1)	1.19 (0.81 - 1.74)	0.378
Walk for at least 30 minutes daily								
Yes	1016	449 (44.2)	1.0		1018	636 (62.5)	1.0	
No	314	151 (48.1)	1.17 (0.91 - 1.51)	0.225	314	209 (66.5)	1.19 (0.91- 1.56)	0.189
Hypertension (BP ≥140/90 mm Hg)								
No	955	367 (38.4)	1.0		956	537 (56.2)	1.0	
Yes	418	248 (59.3)	2.33 (1.84- 2.95)	0.000	419	335 (79.9)	3.11 (2.37 - 4.08)	0.000
Diabetic								
No	1260	556 (44.1)	1.0		1261	775 (61.5)	1.0	
Yes	113	59 (52.2)	1.38 (0.94 - 2.03)	0.100	114	97 (85.1)	3.58 (2.11 - 6.06)	0.000

Table 3 shows the results of multivariate adjusted analyses. The factors independently and significantly associated with being overweight or obese included working as a driver, shop keeper or tailor, having a college or university level education, living in UAE for 6 to 10 years or more than 10 years and having hypertension. The factors independently and significantly associated with central obesity included greater age, working as a driver, businessman or shop-keeper, living in UAE for 6 to 10 years or more than 10 years, and having hypertension. The prevalence of overweight and obesity was extremely high among males who had been living in the UAE for more than five years and working in sedentary occupations (overall 77.4%; 75.0% among drivers, 86.7% among shop keepers and business men, 70.6% among tailors). In addition, the prevalence of central obesity was also high in this group (overall 87.5%; 86.9% among drivers, 93.3% among shopkeepers and business men, 82.3% among tailors). We also evaluated the effects of acculturation on hypertension, smoking and diabetes (statistical analyses are not shown).

**Table 3:** **Multivariable logistic regression: adjusted odds ratios of factors associated with Overweight/obesity and Central obesity in male South Asian immigrants (n = 1,375) Al Ain, Abu Dhabi, UAE, 2012**

	**Overweight AOR**	***p*** **value**	**Central obesity AOR**	***p*** **value**
Nationality	(95% CI)		(95% CI)	
India	0.91 (0.65 - 1.27)	0.602	0.88 (0.62 - 1.27)	0.483
Pakistan	1.48 (0.98 - 2.24)	0.066	1.06 (0.73 - 1.55)	0.747
Bangladesh	1.0		1.0	
Age, Years	1.02 (0.99 - 1.04)	0.065	1.06 (1.03 -1.08)	<0.001
Monthly salary in UAE Dirhams (AED)	1.00 (0.99 - 1.00)	0.735	0.99 (0.99 - 1.00)	0.598
Education				
None	1.0		1.0	
Primary or middle	1.51 (0.95 - 2.41)	0.083	1.58 (0.98 - 2.55)	0.058
Secondary or high school	1.54 (0.96 - 2.49)	0.075	1.27 (0.78 - 2.05)	0.340
College or university	2.05 (1.03 - 4.06	0.040	1.09 (0.55 - 2.23)	0.808
Occupation				
Driver	2.54 (1.65 - 3.89)	<0.001	1.57 (0.99 - 2.48)	0.051
Laborer	1.0		1.21 (0.76 - 1.94)	0.416
Construction worker	1.08 (0.66 - 1.78)	0.745	1.29 (0.76 - 2.20)	0.333
Agriculture worker	1.16 (0.73 - 1.83)	0.523	1.0	
Salesman	1.45 (0.76 - 2.74)	0.253	1.81 (0.87 - 3.75)	0.110
Professional, office worker	1.85 (0.96 - 3.56)	0.066	2.14 (1.02 - 4.47)	0.043
Business man/shop keeper	2.35 (1.08 - 5.08)	0.030	3.05 (1.20 - 7.73)	0.019
Hospitality worker	1.58 (0.82 - 3.02)	0.170	1.38 (0.68 -2.79)	0.361
Tailor	3.33 (1.63 - 6.83)	<0.001	1.98 (0.89 - 4.36)	0.091
Other	1.18 (0.52 - 2.68)	0.687	1.14 (0.46 - 2.83)	0.764
Type of accommodation				
Shared with non-relatives	1.20 (0.78 - 1.83)	0.339	1.07 (0.70 - 1.64)	0.737
Shared with family	1.45 (0.80 - 2.63)	0.213	0.78 (0.42 - 1.45)	0.434
Single accommodation	1.69 (0.97 - 2.94)	0.062	1.43 (0.78 - 2.62)	0.246
Live with sponsor	1.02 (0.63 - 1.73)	0.932	0.78 (0.45 - 1.34)	0.377
Live in a labor camp	1.0		1.0	
Home country setting				
Rural	1.0		1.0	
Urban or semi-urban	1.23 (0.91 - 1.64)	0.170	1.05 (0.76 - 1.46)	0.731
Length of stay in UAE				
1 to 5 years	1.0		1.0	
6 to 10 years	1.81 (1.28 - 2.57)	<0.001	1.63 (1.13 - 2.35)	0.008
>10 years	1.69 (1.15 - 2.48)	0.007	1.95 (1.26 - 3.01)	0.002
Marital status				
Single	1.0		1.0	
Married	1.35 (0.96 - 1.90)	0.080	1.34 (0.95 - 1.88)	0.086
Hypertension (BP ≥140/90 mm Hg)				
No	1.0		1.0	
Yes	1.87 (1.40 - 2.48)	<0.001	2.07 (1.49 - 2.87)	<0.001
Diabetes				
No	1.0		1.0	
Yes	0.78 (0.52 - 1.55)	0.196	1.05 (0.44, 1.89)	0.671

We did not find a significant (p < 0.05) association of hypertension and cigarette smoking with living in the UAE for 6 to 10 years or more than 10 years. Diabetes was significantly associated with 10 or more years of residency in UAE. We did not find a significant relation of other lifestyle variables such as physical activity with duration of residency in UAE.

## Discussion

The prevalence of overweight and obesity in our sample of South Asian immigrants living in the UAE was higher than published estimates from their home countries of India, Pakistan and Bangladesh. The mean BMI in our study was considerably higher compared to working men aged 20 to 59 years in India (31.5 kg/m^2^ versus 23.1 kg/m^2^) [25] and Bangladesh (26.2 kg/m^2^ versus 19.7 kg/m^2^) [26]. Prevalence of overweight and obesity in Pakistani immigrants was more than double compared to their counterparts in Pakistan (50.5% versus 22.0%) [27]. While the prevalence of overweight and obesity among South Asian immigrants living in UAE for more than 10 years (62.7%) was substantially higher than the prevalence amongst their own non-migrant countrymen, obesity was relatively close to age-standardised prevalence rate for the native Emirati population (67.7%) [28].

The prevalence of central obesity (63.4%) in the study sample was higher compared to native Emirati men (58.6%) [28], which is similar to results from other studies reporting high rates of central obesity (classified using the waist-to-hip ratio) among South Asian immigrants [28,29]. Central obesity is an independent risk factor for cardiovascular disease in the Indian subcontinent male population [30]; this highlights the importance of measuring central obesity rather than body mass and height alone.

In our study population, the prevalence of overweight and obesity was significantly higher among those who had been living in the UAE for a longer period. Indeed, the prevalence of overweight and obesity increased from 34.8% with five years residency to 59.3% with 6 to 10 years residency and 62.3% with more than 10 years of residency. Previous studies indicate relatively lower rates of poor health among newly arrived South Asian immigrants in Europe and Canada compared to the general population [31]. However, it would appear that that this “immigrant health advantage” over the native population will decrease as the number of years lived in the host country rises [32,33]. Significant changes in diet amongst immigrants have been reported, with a substantial increase in overall energy and fat intake due to the accessibility and availability of energy-dense foods and snacks. Moreover, there is a switch from whole grains and pulses to more refined sources of carbohydrate along with increased consumption of soft drinks [32]. Our results are consistent with previous studies in which long-term immigrants reported higher rates of obesity, diabetes, hypertension and smoking compared to natives [34]. Study findings provide more evidence on the notion of a *Healthy Migrant Effect* seen in Western countries where upon arrival, immigrants are healthier than the native population, however, their health status declines as the duration of their stay increases [31,32,34]. The UAE has been previously defined as an obesogenic environment due to an accelerated nutrition transition moving away from traditional “healthy” foods and a high level of physical activity to an abundance of “unhealthy” energy-dense foods coupled with a sedentary life style [13]. The prolonged positive energy balance required to elicit high rates of obesity among South Asian immigrant males living in the UAE for five or more years may be due to dietary acculturation characterized by adopting affordable low quality food with high energy intake (primarily from refined sugar and saturated fat) with or without a concomitant reduction in daily energy expenditure (physical activity). It might be the reason for a positive association between diabetes and living in UAE for more than ten years.

Drivers, shopkeepers and tailors reported very low levels of moderate and vigorous physical activity. The prevalence of BMI-derived overweight and obesity, and central obesity estimates was very high among migrants working in sedentary occupations such as drivers, shop keepers and tailors, particularly those who had lived in the UAE for more than five years. These findings are in agreement with other studies that have reported low physical activity and high engagement in sedentary behaviour among South Asian immigrants [35].

There was a significant positive significant association between obesity and college or university level education. The direct association between income and education in developed countries may indicate healthier lifestyle choices in those with higher literacy, particularly those with higher health literacy [36]. Further research is needed to elucidate whether this discrepancy may be due to factors such as poorer health literacy level [37].

An alarming finding is that almost 76% of study participants with hypertension and 44% with diabetes were not aware of their conditions. Compared to white Caucasians, South Asians report overall lower disease awareness: language barriers, religious and sociocultural factors have been attributed to poor awareness and late diagnosis [37,38]. Therefore, the treatment and control of hypertension and diabetes is crucial for reducing cardiovascular death rates as the study population is already prone to developing coronary heart disease at a younger age (<40 years in men) [5,6,8]. Greater emphasis on improving primary health care and health education in this population may increase awareness and promote healthy lifestyles has the potential to improve cardiovascular health.

### Strengths and limitations

There are several strengths of this study. At present, to the best of our knowledge, this is the first study documenting the burden of obesity in South Asian male immigrants living in the UAE. We recruited a large random representative sample (with a good response rate 76.4%) from the mandatory health visa screening centre in the second largest city in the emirate of Abu Dhabi in the UAE. In addition, we utilized an adapted version of the WHO STEPwise questionnaire for NCD risk factor surveillance, two anthropometric indices to classify obesity in our population (BMI and WHR) and objectively measured blood pressure, and also HbAlc (in a sub-sample of our study population). However, there are some limitations inherent in the study design which means the data should be interpreted with caution. Firstly, as with any cross sectional design, it is not possible to draw firm conclusions about the temporal or causal nature of the association between acculturation, various risk factors and obesity. Secondly, we did not use a standardized acculturation scale; rather, we used years of residency as a surrogate measure. Currently, there are no validated multidimensional instruments to measure acculturation among South Asians in the UAE. Future studies may want to consider employing modified versions of standardized and validated acculturation scales that have been used in South Asian immigrant populations in the United Kingdom [39] and the United States [40]. Alternatively, researchers could develop a contextual- and culturally-specific acculturation scale for use in migrant populations in the UAE that would include specific acculturation domains such as dietary preferences, clothing style, use of own-culture media, language use and fluency, social connections, and cultural/religious beliefs. Thirdly, we recruited the sample in the visa screening center in Al Ain, Abu Dhabi emirate and the study sample may thus not represent the South Asian male population residing in other emirates in the UAE. However, we would not expect the socioeconomic and lifestyle characteristics of our sample to differ greatly from South Asians in other emirates. The large sample size provides sufficient power to evaluate differences in the dependent variables by socio-demographic and lifestyle variables. Finally, it should be noted that our study results may be specific to migrant men and may not translate to South Asian women.

## Conclusions

This is the first study to reveal a high prevalence of overweight, obesity, hypertension and diabetes among a relatively young South Asian male immigrant population living in the UAE. Study findings suggest a diminished ‘Healthy Migrant Effect’ with increased years of residency possibly due to greater acculturation (particularly greater than five years residency) and a transition in lifestyle behaviors. Preventive health measures are often ignored in this population. Culturally-tailored health education interventions targeting the maintenance of a healthy body size, coupled with regular assessments of glucose control and blood pressure are urgently required in this population. The design, development, and implementation of future health promotion campaigns, interventions, and strategies amongst South Asians must consider the sociocultural, religious, ethnic, and educational diversity of this population.

## Abbreviations

AED: Emirati Dirham
BMI: Body mass index
CI: Confidence interval
HbA1c: Glycated hemoglobin
NCD: Non-communicable chronic diseases
UAE: United Arab Emirates
USD: United States Dollar
WHO: World Health Organization
WC: Waist circumference
WHR: Waist-to-hip ratio
